# The Prognostic Value of ^18^F-FDG PET/CT and KRAS Mutation in Colorectal Cancers

**DOI:** 10.4274/mirt.galenos.2019.33866

**Published:** 2020-02-17

**Authors:** Esra Arslan, Tamer Aksoy, Rıza Umar Gürsu, Nevra Dursun, Ekrem Çakar, Tevfik Fikret Çermik

**Affiliations:** 1 University of Health and Sciences, İstanbul Training and Research Hospital, Clinic of Nuclear Medicine, İstanbul, Turkey; 2 University of Health and Sciences, İstanbul Training and Research Hospital, Clinic of Medical Oncology, İstanbul, Turkey; 3 University of Health and Sciences, İstanbul Training and Research Hospital, Clinic of Pathology, İstanbul, Turkey; 4 University of Health and Sciences, İstanbul Training and Research Hospital, Clinic of Surgery, İstanbul, Turkey

**Keywords:** Colorectal cancer, KRAS mutation, 18F-fluoro-deoxy-glucose positron emission tomography/computerized tomography (18F-FDG PET/ CT), prognosis

## Abstract

**Objective:**

Prognostic effect of KRAS mutation and side of tumor in colorectal cancer is a highly controversial subject. Therefore, we evaluated the association between FDG uptake pattern in ^18^F-fluoro-2-deoxy-glucose positron emission tomography/computed tomography (^18^F-FDG PET/CT) imaging and KRAS mutation and tumor localization in patients with a diagnosis of colon cancer and assessed the effects of these three factors on prognosis and survival.

**Methods:**

Eighty-three patients with colorectal cancer were retrospectively included in this study. ^18^F-FDG PET/CT study was performed for pretreatment staging. The maximum standardized uptake value (SUV_max_) of the primary tumor and survival data of patients were compared between groups. KRAS mutations were detected with the help of real-time Polymerase Chain Reaction technique through genomic DNA extracted from paraffin-embedded tumor tissue blocks. Tumor lesions with potential KRAS mutations were classified as mutant KRAS and wild type.

**Results:**

Twenty five patients were female while 58 were male. The mean age of the patients was 59.8±11.3 years. Mean follow-up was 35.5±18.9 months. Primary tumor was localized in the left colon in 83.1% of patients and in the right colon in 16.9%. KRAS mutation was detected in 54.2% (n=45) of patients. Mean SUV_max_ of patients with primary tumor was estimated to be 21.1±9.1 (range= 6.0-47.5). Mean tumor SUV_max_ of patients with a KRAS mutation (24.0±9.0) was found to be significantly higher than those without KRAS mutation (17.7±8.2) (p=0.001). Mean survival was significantly shorter in patients with locoregional nodal metastasis than in patients without locoregional nodal metastasis as well as in patients with distant nodal metastasis than in patients without distant nodal metastasis and in patients with organ metastasis in initial PET/CT than in patients without organ metastasis. Also, mean survival was nearly statistically-significantly shorter in patients with tumors located in left colon (34.2±19.4) than in right colon (43.2±14.6) (p=0.059). However, we found no significant impact of KRAS mutation on survival.

**Conclusion:**

In our study, we found that tumor localization had no significant effect on prognosis in patients with colon cancer. On the other hand, FDG uptake was observed to be higher in the presence of KRAS mutation and it was concluded that coexistence of KRAS mutation with higher SUV_max_ is a negative prognostic factor.

## Abstract (tr)

**Amaç:**

Kolorektal kanserde KRAS mutasyonu ve tümörün lokalizasyonu ile ilişkili prognostik etki tartışmalı bir konudur. Bu nedenle ^18^F-floro-2-deoksi-glukoz pozitron emisyon tomografi/bilgisayarlı tomografi (^18^F-FDG PET/BT) görüntülemede FDG tutulum paterni ile kolon kanseri tanısı almış hastalarda KRAS mutasyonu ve tümör lokalizasyonu arasındaki ilişkiyi değerlendirdik. Bu üç faktörün prognoz ve sağkalım üzerindeki etkileri değerlendirildi.

**Yöntem:**

Kolorektal kanser tanılı 83 hasta retrospektif olarak bu çalışmaya dahil edildi. Tedavi öncesi evreleme için ^18^F-FDG PET/BT çalışması yapıldı. Primer tümöre ait ortalama standart tutulum değeri (SUV_maks_) ve sağkalım verileri gruplar arasında karşılaştırıldı. KRAS mutasyonları, parafine gömülü tümör dokusu bloklarından ekstrakte edilen genomik DNA ile gerçek zamanlı polimeraz zincir reaksiyonu tekniği ile tespit edildi. Potansiyel KRAS mutasyonları olan tümöral lezyonlar mutant KRAS ve wild tip olarak sınıflandırıldı.

**Bulgular:**

Olguların 25’i kadın, 58’i erkekti. Hastaların yaş ortalaması 59,8±11,3 idi. Ortalama takip süresi 35,5±18,9 aydı. Primer tümör hastaların %83,1’inde sol kolonda ve %16,9’unda sağ kolonda lokalize idi. KRAS mutasyonu hastaların %54,2’sinde (n=45) tespit edildi. Primer tümörün ortalama SUV_maks_ değeri 21,1±9,1 (dağılım aralığı= 6,0-47,5) olarak hesaplandı. KRAS mutasyonu olan hastaların ortalama tümör SUV_maks_’ı (24,0±9,0), KRAS mutasyonu olmayanlara (17,7±8,2) göre anlamlı derecede yüksek bulundu (p=0,001). Ortalama sağkalım, lokorejyonel nodal metastazı olan hastalarda, lokorejyonel nodal metastazı olmayanlara göre, uzak nodal metastazı olan hastalarda, uzak nodal metastazı olmayanlara göre ve evreleme amaçlı PET/BT’de organ metastazı olanlarda, organ metastazı olmayanlara göre anlamlı olarak daha kısaydı. Ayrıca, ortalama sağkalım sol kolona lokalize tümörü bulunan hastalarda (34,2±19,4) sağ kolona lokalize tümörü bulunanlara göre (43,2±14,6) istatistiksel olarak anlamlı derecede kısaydı (p=0,059). Ancak KRAS mutasyonunun sağkalım üzerinde anlamlı bir etkisi olmadığı belirlendi.

**Sonuç:**

Çalışmamızda, kolon tümörü lokalizasyonunun prognoz üzerinde anlamlı bir etkisinin olmadığını bulduk. Öte yandan, FDG tutulumunun KRAS mutasyonu varlığında daha yüksek olduğu gözlendi ve KRAS mutasyonunun yüksek SUV_maks_ ile birlikteliğinin negatif prognostik bir faktör olduğu sonucuna varıldı.

## Introduction

Colorectal cancer is the third most common malignancy in males and the second most common in females worldwide (1). The disease has high incidence and mortality rates in countries with strong economies while its incidence is reported to be rising in developing countries (2). Western diet is held responsible for this higher incidence. In the 2011 update, the American Institute for Cancer Research reported that red and processed meat is also associated with the increasing incidence of colon cancer (3,4).

Stage, grade, presence of obstruction and/or perforation and presence of vascular invasion, nodal and organ metastases alter the prognosis in colon cancer. In addition, RAS mutation, surgical intervention, radiation therapy and adjuvant chemotherapy methods are also prognostic factors (5,6). It is well known that a group of prominent genetic mutations along with the accumulation of environmental risk factors increase the transformation into colorectal cancers (5). The RAS gene family (KRAS, HRAS and NRAS) codes membrane-bound G proteins (p21^RAS^) that regulate cell growth and apoptosis via endothelial growth factor receptor (7). KRAS is the most commonly mutated oncogene associated with pancreatic, colorectal and pulmonary malignancies. As a result of deleted KRAS allele, the mutant p21^KRAS^ is activated and induces cell proliferation. Therefore, studies have recently concentrated on KRAS-targeted therapies (8).

It is difficult to establish the association between prognosis and treatment options in patients with colorectal cancer. Heterogeneous survival data are reported even in patients with same pathological grade. Therefore, establishing the prognostic factors accurately is vital in determining high risk patients (9). However, certain contexts are still controversial for prognosis. For example, there are contradictory prognosis and survival data published on right and left colon which arise from different anatomical and embryonic origins (10). The findings of studies that evaluate right and left colon difference along with the presence of RAS mutation are particularly limited and controversial (11). Therefore, in our study, we evaluated FDG uptake pattern in patients who were initially staged with ^18^F-fluoro-2-deoxy-glucose positron emission tomography/computed tomography (^18^F-FDG PET/CT) imaging, which is increasingly used in the diagnosis, staging and prognosis determination of various types of cancer, and the presence of KRAS mutation and the association of tumor localization with these two factors and questioned the prognostic power and survival impact of these associations.

## Materials and Methods

### Patients

Eighty-three patients with colorectal cancer who had staging and follow-up ^18^F-FDG PET/CT examinations and tumor specimen for mutation analysis between September 2012 and June 2018 were included in this retrospective study. Histopathologic diagnosis and ^18^F-FDG PET/CT imaging were obtained prior to surgical resection and/or chemotherapy/radiation therapy. The study was approved by the İstanbul Training and Research Hospital Local Ethics Committee (no: 2018/1228). The diagnosis and histopathologic analysis of primary colorectal cancer were verified with materials obtained by surgery or from the biopsy. Staging was performed according to Tumor, Node and Metastases (TNM) staging system for colon cancer in concordance with the American Joint Committee on Cancer Guidelines (12).

### ^18^F-FDG PET/CT Imaging

In our study, primary lesions were assessed according to the location (left or right colon), ^18^F-FDG uptake and the presence of lymph node and distant metastases in PET/CT. Patients whose blood glucose levels were below 150 mg/dL after six hours of fasting were suitable for the procedure. Sixty minutes after the intravenous injection of 3.7-5.2 MBq/kg ^18^F-FDG, PET/CT imaging was obtained from vertex to upper femur (Biograph mCT ultra HD LSO PET/CT; Siemens Molecular Imaging; Hoffman Estates, IL, USA). CT imaging for PET/CT was performed using a multi-detector scanner with 20 slices, at 80-140 kV, 20-266 mAs, 0.8 pitch and 512x512 matrix [personalized settings determined by automatic exposure control system; automatically defined by the software used by manufacturer (CareDose 4D) depending on the patient and region assessed]. CT imaging was performed between vertex and upper-thigh in craniocaudal direction with 5 mm of slice thickness and 0.5 seconds of rotation time. Then, PET imaging was performed in the same range through craniocaudal direction at 8 to 9 bed positions, 1.5 minutes for each PET bed using LSO PET scanner. Ultra HD images were acquired using time of flight (TOF) + true X algorithm at iteration 2 and subset 16 values for reconstruction.

Standardized uptake value (SUV_max_) was calculated by drawing a region of interest (ROI) around the region with the highest ^18^F-FDG uptake. SUV_max_ was calculated automatically by the software using the following formula: maximum activity within ROI (MBq/mL) /injected ^18^F-FDG dose (MBq/kg).

### Mutation Analysis

DNA, for the assessment of KRAS mutation test, was extracted from paraffin-embedded tumor tissue blocks (Qiagen, Hilden, Germany), which obtained from biopsy materials after primary tumor resection or biopsy. Polymerase chain reaction was performed via some extraction, incubation and amplifications cycles according to the manufacturer’s instructions. Tumor lesions with potential KRAS mutations in

### Codon 12;

+ ___ Gly12Asp (GGT>GAT)

+ ___ Gly12Val (GGT>GTT)

+ ___ Gly12Cys (GGT>TGT)

+ ___ Gly12Ser (GGT>AGT)

+ ___ Gly12Ala (GGT>GCT)

+ ___ Gly12 Arg (GGT>CGT)

+ ___ Codon 12 mutation, not otherwise specified,

### Codon 13;

+ ___ Gly13Asp (GGC>GAC)

+ ___ Gly13Arg (GGC>CGC)

+ ___ Gly13Cys (GGC>TGC)

+ ___ Gly13Ala (GGC>GCC)

+ ___ Gly13Val (GGC>GTC)

+ ___ Codon 13 mutation, not otherwise specified,

### Codon 59,

### Codon 61;

+ ___ Gln61Leu (CAA>CTA)

+ ___ Gln61His (CAA>CAC)

+ ___ Codon 61 mutation, not otherwise specified,

### Codon 117,

### Codon 146;

+ ___ Ala146Thr (G436A) (GCA>ACA)

+ ___ Codon 146 mutation, not otherwise specified was detected.

### Statistical Analysis

All data were analyzed using SPSS software for Windows (v21.0; IBM, Armonk, NY, USA). Data were expressed as mean and standard deviation, median (min-max), distribution frequencies and percentages, when appropriate. Normalization of data distribution was evaluated using Kolmogorov-Smirnov test. For variables that were not normally-distributed, comparison was performed using Mann-Whitney U and Kruskal-Wallis tests while correlation was evaluated using Pearson’s test. Categorical variables were evaluated using chi-square test. Survival rates were evaluated with Kaplan-Meier analysis. P values <0.05 were considered statistically significant.

## Results

**Figure 1:**
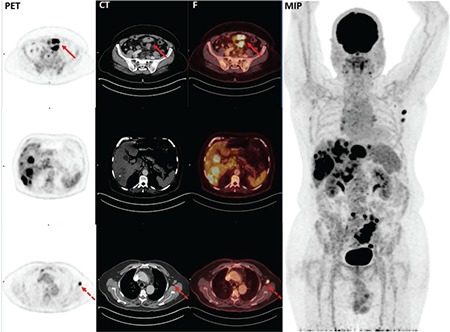
A 65-year-old male patient with mutant type adenocarcinoma localized to sigmoid colon positron emission tomography, computed tomography, fusion, maximum intensity projection. Red arrows show fluorodeoxyglucose uptake of the primary tumor, primary tumor: SUV_max_: 31.5 with multiple liver metastases. Dotted red arrows show left axillary lymph node metastases. SUV_max_: Maximum standardized uptake value, PET: Positron emission tomography, CT: Computed tomography, F: Fusion, MIP: Maximum intensity projection

**Figure 2:**
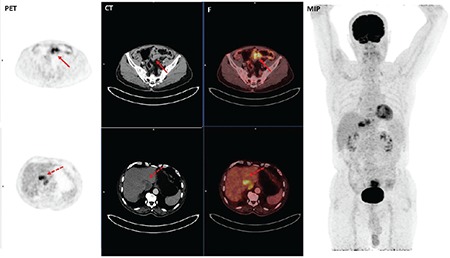
A 70-year-old male patient with wild type adenocarcinoma localized to sigmoid colon positron emission tomography, computed tomography, Fusion, maximum intensity projection. Red arrows show fluorodeoxyglucose uptake of the primary tumor. Primary tumor: SUV_max_: 11.5 dotted red arrows show hepatic metastases SUV_max_: Maximum standardized uptake value, PET: Positron emission tomography, CT: Computed tomography, F: Fusion, MIP: Maximum intensity projection

Out of 83 subjects with a diagnosis of colorectal cancer, 25 (30.1%) were female and 58 (69.9%) were male. Mean age was 59.8±11.3 years (range=35-81). Sixty-nine (83.1%) tumoral lesions were located in the left colon while 14 (16.9%) were located in the right colon. KRAS mutation was found in 54.2% (n=45) of patients. Thirty-eight (84.4%) of KRAS mutant colorectal tumors were left-sided while 7 (15.6%) were right-sided. Also, ^18^F-FDG uptake was observed in all primary lesions (n=83) and mean SUV_max_ was estimated to be 21.1±9.2 (median=20.2, range=6.0-47.5). When clinical characteristics of the patients were considered along with ^18^F-FDG uptake, no statistically significant association was found between the mean SUV_max_ in the group with patients younger than 50 years (19.6±6.2) (n=18) and the mean SUV_max_ in the group with patients ≥50 years (21.5±9.8) (n=65) (p=0.436). Similarly, patient gender had no statistically significant impact on primary tumor mean SUV_max_ (p=0.452) (Table 1). When subjects were evaluated for tumor localization, there was no difference between left-sided (21.2±8.6) and right-sided (20.4±11.6) tumors in terms of mean SUV_max_ (p=0.768). Mean tumor SUV_max_ of patients with KRAS mutation was 24.0±9.0 (Figure 1) while this value was calculated as 17.7±8.2 (Figure 2) in patients without mutation (wild type). Therefore, mean SUV_max_ in subjects with KRAS mutation was significantly higher when compared to wild type (p=0.001) (Table 1).

According  to the staging 1 patient had stage II, 13 patients had stage III, and 69 patients had stage IV disease according to The American Joint Committee on Cancer TNM classification and staging system. Sixty-eight (81.9%) subjects were found to have locoregional nodal metastases at the time of diagnosis; 54 (79%) of these tumors were left-sided and 14 (21%) were right-sided. Twenty-six (31.3%) subjects had distant nodal metastases, 20 (77%) of which were left-sided and 6 (23%) of which were right-sided colon tumors. There was no difference between subjects with locoregional or distant nodal metastases and subjects without nodal metastases in terms of mean tumor SUV_max_ (p=0.928 and 0.135, respectively) (Table 1). Initial PET/CT revealed organ metastases in 81.9% (n=68) of patients, while distant metastases developed later in follow-up in 18.1% (n=15). Similarly, there was no statistically significant difference between the group with distant metastases at diagnosis and the group with metastases developed in follow-up in terms of primary tumor mean SUV_max_ (p=0.323). Distant organ metastases were most frequently observed in liver (47.0%, n=39), followed by lung (19.3%, n=16), peritoneum (8.4% ,n=7) and liver + peritoneum (4.8%, n=4). Of patients, 20.5% (n=17) had multiorgan metastases.

Mean follow-up period of patients included was 35.5±18.9 months (range=3.8-73.4 months). Comparison of mean survival and clinical characteristics is presented in Table 2. According to this, mean survival time of patients with locoregional nodal metastasis and distant nodal metastasis (33.4±18.2 and 29.8±15.6 months, respectively) was significantly shorter than the survival time of patients without locoregional nodal metastasis and distant nodal metastasis (33.4±18.2 and 29.8±15.6 months, respectively) (p=0.037 and 0.046, respectively) (log rank=0.020 and 0.001, respectively) (Graphic 1, 2). Also, mean survival time of patients with organ metastasis at diagnosis (33.4±19.0 months) was observed to be significantly shorter than patients who developed distant metastasis during follow-up (44.7±16.1 month) (p=0.037, log rank=0.023) (Graphic 3). Mean survival was nearly statistically-significantly shorter in patients with tumors located to left colon (34.2±19.4) than in right colon (43.2±14.6) (p=0.059). KRAS mutation, age and sex were found to have no statistically significant influence on mean survival (p=0.136, 0.224 and 0.257, respectively).

Mean survival time after diagnosis was estimated to be 17.4±13.8 months (range=1.3-62.2 months). Mean overall survival after diagnosis of patients with KRAS-mutant and wild-type tumors was 18.3±16.3 and 16. 7±11.5 months, respectively. Also, mean overall survival of patients with primary tumor located to left and right colon was 18.8±14.9 and 12.6±7.3 months, respectively with no statistically significant difference between groups (p values=0.818 and 0.391, respectively).

In our study, 52 patients (62.7%) received bevacizumab, 13 patients (15.7%) received cetuximab and 8 patients (9.6%) received panitumumab protocol as primary treatment. Of patients 31.3% (n=26) showed progression and 6 of these (7.2%) received cetuximab, 17 (20.5%) received bevacizumab and 1 (1.2%) received aflibercept as second-line treatment. The presence of KRAS mutation and tumor localization were not significantly associated with progression (p=0.603 and 0.687, respectively). At the end of follow-up period, 57.8% of patients (n=48) were deceased. Among survivors, 7 patients (8.4%) were in remission, 20 patients were refractory and 8 were lost to follow-up (9.6%). The presence of KRAS mutation and tumor localization were not significantly associated with mortality (p=0.073 and 0.563, respectively).

## Discussion

Despite the globally rising prevalence of colorectal cancers, recent developments in early diagnosis and treatment options have caused a decline in its incidence in especially advanced ages. However, the increase in the incidence of colorectal cancers in patients younger than 50 years is noteworthy (1,2,3,4). Because poor prognostic factors tend to accumulate in younger patients, predictive value of prognostic factors gain importance (9). Increased FDG uptake in ^18^F-FDG PET/CT imaging, which has been widely used in the diagnosis and staging of many malignancies including breast, gastric and lung carcinoma as well as colorectal cancer, is significantly associated with aggressive tumor pattern and poor prognosis (13,14,15,16). Bundschuh et al. (17) evaluated 27 patients with locally advanced rectal cancer and reported that ^18^F-FDG PET/CT imaging has significant prognostic advantage in evaluating disease progression and provides important data in assessing treatment response and determining patients with high risk. In their study with 67 patients with colorectal cancer, Petersen et al. (18) stated the importance of ^18^F-FDG PET/CT imaging in staging and underlined that it was possible to change treatment strategy in approximately 30% of patients with the contribution of ^18^F-FDG PET/CT imaging. Besides these, the most important factors determining the prognosis in colorectal cancers are certainly the presence of nodal metastases and distant organ metastases. However, there are also prognostic factors specific to colorectal cancers which are sometimes controversial. The leading is KRAS mutations (8,9). Roth et al. (19) evaluated the prognostic effects of KRAS and BRAF mutations by extracting DNA from 1321 of 1404 specimens of colon cancer. The researcher concluded that KRAS mutation had no major prognostic value in patients with stage II and III colon cancer and did not influence survival. Ogino et al. (20) found similar results and reported that KRAS mutation had no effect on prognosis or survival. On the contrary, Lee et al. (21) found that KRAS mutations had negative prognostic effects on 437 patients with stage II and III colon cancer. Vogelaar et al. (22) pointed out the association between the presence of KRAS mutation and shorter survival. Similarly, Ribeiro et al. (23) evaluated 58 patients with metastatic colon adenocarcinoma and reported that KRAS mutation was significantly associated with lymph node metastasis and organ metastasis when evaluated together with CD44 and CD166 expression. As the literature data indicate, findings about KRAS are quite contradictory. In our study, we observed that survival times shortened significantly with locoregional and distant nodal metastasis and the presence of distant organ metastasis at diagnosis while KRAS mutation had no statistically significant effect on mean survival. Also, the presence of KRAS mutation was found to have no significant impact on patients who were deceased or showed progression after treatment. However, when KRAS mutation was assessed together with FDG uptake pattern, mean tumor SUV_max_ was observed to increase significantly in subjects with KRAS mutation. Therefore, considering that increased FDG uptake is associated with aggressive tumor characteristics and negative prognostic effect, increased SUV_max_ in the presence of KRAS mutations was interpreted as a poor prognostic factor.

Another controversial topic in prognostic factor assessment for colon cancer is tumor side. Embryologic, histological, genetic and immunological differences of the right and left colon provide basis for these discussion. Certain studies in the literature could not define any difference between the right and left colon that can affect survival, while others reported the left colon to be associated with higher survival (10). Karim et al. (24) evaluated 6365 patients who were diagnosed with early stage colon cancer between 2002 and 2008 and concluded that tumor side had no significant association with overall survival or cancer-specific survival. In contrast, Petrelli et al. (25) conducted a meta-analysis that includes 66 studies with 1.437.846 patients and reported that tumors localized to left colon had significantly lower risk of death and this was independent of stage, ethnicity and adjuvant chemotherapy. Similarly, in the study by Ulanja et al. (10) that included 163,986 patients with colon cancer with the help of the “Surveillance, Epidemiology, and End Results” database, the researchers reported that 52.3% of tumors were localized in the right colon and 47.7% were in the left colon while left colon cancer was significantly associated with better survival. In our study, 83.1% of tumoral lesions were left-sided while 16.9% were right-sided. We found no statistically significant difference between tumors localized to right or left colon both in terms of FDG uptake and survival. Also, tumor localization and the presence of KRAS mutation had no statistically significant impact on mortality. However, we think that our prognostic evaluation using both ^18^F-FDG PET/CT imaging modality and the presence of KRAS mutation may have a predictive value.

## Conclusion

In conclusion, side of tumor in patients with colorectal cancer was not found to have significant impact on prognosis in our study. On the other hand, we observed an association between the presence of KRAS mutation and increased FDG uptake in the primary tumor. We think that this association may have a predictive value for poor prognosis and our data may contribute to patient management especially in this subgroup.

## Figures and Tables

**Table 1:**
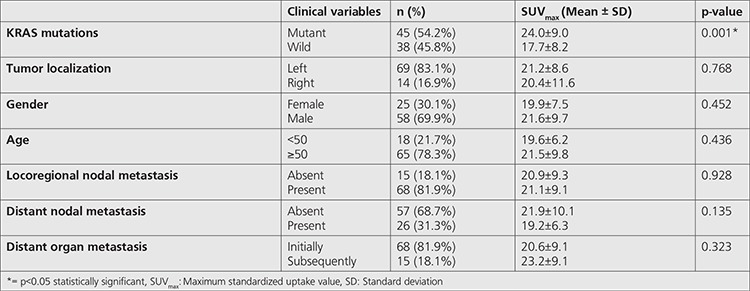
The association between mean SUV_max_ and clinical, histopathological features of patients

**Table 2:**
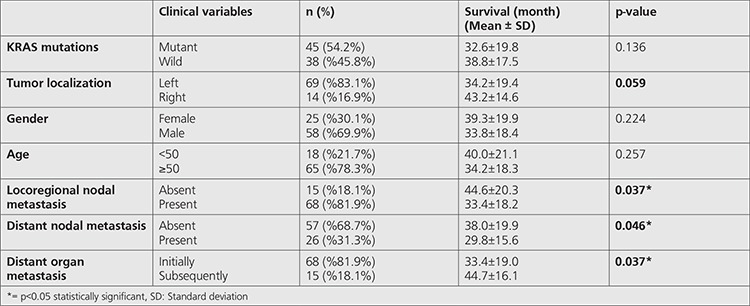
Comparison of patients’ clinical/histopathological features and mean survival times

**Graph 1:**
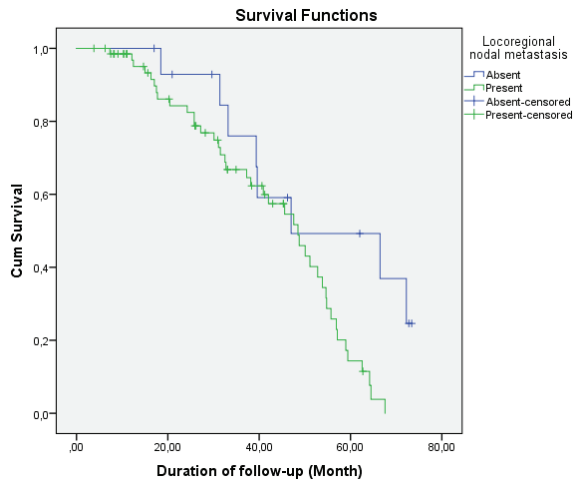
Survival chart according to locoregional nodal involvement (log rank=0.020)

**Graph 2:**
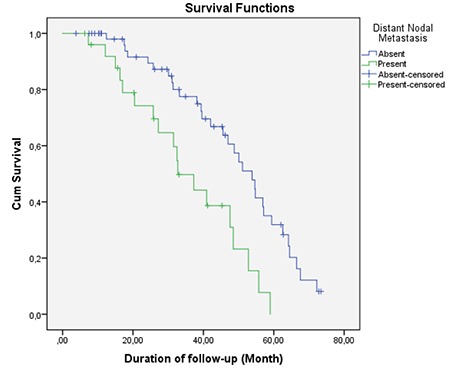
Survival chart according to distant nodal involvement (log rank=0.001)

**Graph 3:**
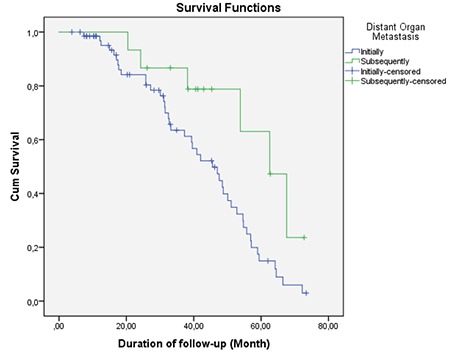
Survival chart according to distant visceral organ metastasis (log rank=0.023)
